# Cost effectiveness of total knee arthroplasty from a health care providers' perspective before and after introduction of an interdisciplinary clinical pathway - is investment always improvement?

**DOI:** 10.1186/1472-6963-11-338

**Published:** 2011-12-14

**Authors:** Frank Krummenauer, Klaus-Peter Guenther, Stephan Kirschner

**Affiliations:** 1 Institute for Medical Biometry and Epidemiology Faculty of Health Sciences, University of Witten/Herdecke, Germany; 2 Department of Orthopedic Surgery University Hospital Carl Gustav Carus Medical Falculty of the Technical University of Dresden, Germany

**Keywords:** Total Knee Arthroplasty (TKA), clinical pathway, WOMAC index, process costs, cost effectiveness

## Abstract

**Background:**

Total knee arthroplasty (TKA) is an effective, but also cost-intensive health care intervention for end stage osteoarthritis. This investigation was designed to evaluate the cost-effectiveness of TKA before versus after introduction of an interdisciplinary clinical pathway from a University Orthopedic Surgery Department's cost perspective as an interdisciplinary full service health care provider.

**Methods:**

A prospective trial recruited two sequential cohorts of 132 and 128 consecutive patients, who were interviewed by means of the WOMAC questionnaire. Direct process costs from the health care providers' perspective were estimated according to the German DRG calculation framework. The health economic evaluation was based on margiual cost-effectveness ratios (MCERs); an individual marginal cost effectiveness relation ≤ 100 € per % WOMAC index increase was considered as primary endpoint of the confirmatory cohort comparison. The interdisciplinary clinical pathway under consideration primarily consisted of a voluntary preoperative personal briefing of patients concerning postoperatively expectable progess in health status and optimum use of walking aids after surgery. All patients were supplied with written information on these topics, attendance of the personal briefing also included preoperative training for postoperative mobilisation by the Department's physiotherapeutic staff.

**Results:**

An individual marginal cost effectiveness relation ≤ 100 €/% WOMAC index increase was found in 38% of the patients in the pre pathway implementation cohort versus in 30% of the post pathway implementation cohort (Fisher p = 0.278). Both cohorts showed substantial improvement in WOMAC scores (39 versus 35% in median), whereas the cohort did not differ significantly in the median WOMAC score before surgery (41% for the pre pathway cohort versus 44% for the post pathway cohort). Despite a locally significant decrease in costs (4303 versus 4194 € in median), the individual cost/benefit relation became worse after introduction of the pathway: for the first cohort the MCER was estimated 108 € per gained % WOMAC index increase (86 - 150 €/%) versus 118 €/% WOMAC gain (93 - 173 €/%) in the second cohort after pathway implementation. In summary, the proposed critical pathway for TKA could be shown to be significantly cost efficient, but not cost effective concerning functional outcome, when the above individual marginal cost effectiveness criterion was concentrated on.

**Conclusions:**

The introduction of an interdisciplinary clinical pathway does not necessarily improve patient related outcomes. On the contrary, cost effectiveness from the health care providers' perspective may even turn out remarkably reduced in the setting considered here (functional outcome assessment after treatment by a full service health care provider).

## Background

Total knee replacement/arthroplasty (TKA) for end stage osteoarthritis is a successful intervention to reduce pain and improve function of the patients [1,2]; as a consequence, in ageing populations the need for total joint arthroplasty is rising, [3] in particular for TKA [4].

As a parallel development clinical pathways have been increasingly considered over the past two decades to improve the management of patients and to control for the costs of medical treatment [5,6]. Different interventions were reported over the last decades to control costs in total joint arthroplasty. Implant standardisation [7-9] and shortening hospital length [10,11] of stay were most often used in clinical pathways.

A recent meta-analysis on the effects of clinical pathways for total joint arthroplasty in terms of length of stay (LOS) as well as expectable changes in re-admission rates reported complication profiles and costs [12]. Interestingly, the observed effects were not unambiguous: no clinically relevant effects could be confirmed for clinical pathways concerning the rate of discharge to home when compared to standard care. On the other hand, this meta-analysis could demonstrate pathways to have beneficial effects on costs, LOS and complication rates. Another recent meta-analysis of the effects of clinical pathways, which was not limited to Orthopedic Surgery, confirmed the positive effect on LOS in particular for invasive procedures [13], although no effects were found for re-admission rates and in-house complication rates. A wide heterogenity between studies reporting LOS data and costs in the context of critical pathway implementation was reported.

The introduction of a clinical pathway does not represent a standardized intervention. By the time being, there is only little research done on methods for the evaluation of clinical pathways [14,15]. Although economic process evaluation methods do offer ideas for analysis of pathway implementations, their design and analysis approach is hardly suitable for the health care evaluation setting: one reason for this is the necessity to simultaneously consider process quality and costs as well as non-process (but rather patient-related) clinical outcome quality indicators. To decide whether a clinical pathway has a positive effect on costs, its putative improvement in terms of patient-related function and quality of life has to be balanced against health care provider cost profiles [16].

The objective of the present study was to evaluate the cost effectiveness of total knee arthroplasty (TKA) with respect to the introduction of an interdisciplinary clinical pathway from the health care providers' perspective under consideration of patient related (functional) outcome indicators. Note that this investigation will not focus on a reduction of the TKA patients' indivdiual or median length of stay as concentrated on by most recent literature: when the investigation was designed for implementation in an Orthopedic Surgery department, it could be assumed, that this department already had implemented standard processes achieving a somewhat short median overall LOS in previous process optimization steps. As a consequence the critical pathway under consideration was designed to rather introduce patient-related features such as preoperative information on postoperatively expectable gain in health and mobility - and thereby to address compliance-related issues rather than the underlying health care process itself. Nevertheless, since introduction of these pathway components had to be fully accomplished by the Orthopedic Surgery Department "at its own costs", the authors decided to retain the Department's perspective for the overall cost effectiveness consideration of implementing the clincal pathway.

## Methods

The primary intention of this investigation was to derive an estimate for the cost effectiveness of TKA before and after introduction of an interdisciplinary clinical pathway into clinical routine. For that purpose a prospective sequential cohort observation with the aim of simultaneously estimating the process costs from a full service (University Hospital) health care providers' perspective and the corresponding patient-related benefit of TKA was implemented.

### Study Design

This prospective cost effectiveness investigation comprised individual data of patients, who underwent unilateral TKA at the Orthopedic Surgery Department of the Dresden University Hospital (Germany) in 2006 and 2007. The Dresden University Hospital's Orthopedic Surgery Department consists of several clinical units, among them a well-established hip and knee arthroplasty unit run by two highly experienced senior Orthopedic surgeons (KPG and SK). The unit has direct access to physiotherapeutic staff and facilities, and thereby can enable to implement improvement concenpts on patient care pathways immediately after identification in direct communication with local nursing and physiotherapy staff.

A total of 260 patients with the clinical indication for TKA were enrolled consecutively and asked for participation in this prospective health economic investigation. After written informed consent patients underwent a written interview by means of the WOMAC questionnaire one week before surgery. The interviews were scheduled by a local study coordinator, who offered assistance to the interviewee if required. Furthermore the patients were invited for a three months recall to undergo the same interview.

**Figure 1:**
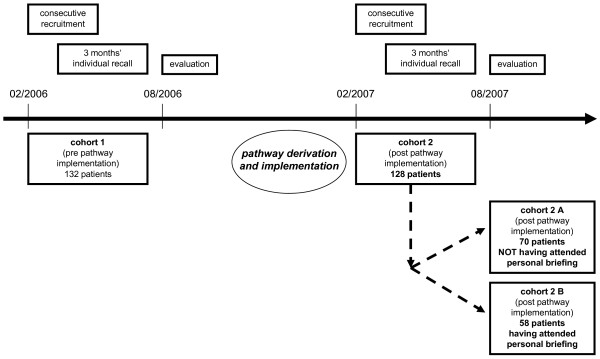
**Illustration of the sequential cohort design first consecutively recruiting patients, who underwent total knee arthroplasty (TKA) before implementation of a critical pathway on TKA, and then recruiting 128 patients, who underwent TKA after pathway implementation (the latter being stratified for attendance of a personal briefing as a voluntary part of the pathway post-hoc during exploratory analysis of the cohorts' clinical data)**.

The overall cohort of 260 patients comprised two sequentially recruited (sub) cohorts: a process documentation of the clinical course within a first cohort of 132 patients was used to derive and then implement an interdisciplinary clinical pathway on TKA; after a three months period of confirming this pathway proposal within clinical routine, a second independent cohort of 128 patients undergoing TKA was recruited and documented at the same hospital. The aim of this sequential cohort design was to compare the cost/benefit relation of TKA within one clinical health care provider unit before and after introduction of an interdisciplinary clinical pathway. Figure 1 summarizes the sequential cohort design, which was ratified by the local Independent Ethics Committee of the Medical Faculty of Dresden Technical University by June 25^th ^2005.

### Surgical Procedure

The study population consisted of patients with primary or secondary osteoarthritis of the knee joint stage 3 or 4 according to Kellgren. The patients were routinely operated under tourniquet after admission of perioperative antibiotic prophylaxis in a laminar airflow room. Through a straight skin incision and a medial parapatellar approach a cemented Natural Knee II (Zimmer, Germany) total knee arthroplasty without resurfacing of the patella was implanted. Postoperatively all patients received a risk adjusted anticoagulation treatment, for example, high risk patients received Arixthra^® ^1 × 1 s.c. injections over six weeks. Postoperatively all patients were allowed for full weight bearing. After discharge from hospital the patients were transferred to an inpatient rehabilitation unit for further three weeks.

### Clinical Pathway Description

The study population was selected from the waiting list for total knee arthroplasty at the trial coordinators' Orthopedic Surgery department. Patients with severe deformities, bone defects, prior infections, posttraumatic disease, instabilities or revisions were not considered for inclusion into the clinical pathway. All patients were seen 5-7 days prior to surgery to check their medical condition and to ensure completeness of the medical records.

Patients treated within the clinical pathway were invited to an information session [17]. About one month prior to surgery the patients were informed about their disease, the surgical procedure and the post surgery stationary course as well as the standard rehabilitation process thereafter by a senior orthopedic surgeon (SK). Further information about anaesthesia and the post surgical pain therapy were presented by a senior anaesthesiologist. During the same schedule patients were educated using crutches and physiotherapy by a physiotherapist. Patients had the opportunity to ask and discuss any given information as well as any further question. The patients received a printed booklet [18] containing information about the orthopaedic procedure and the subsuquent rehabilitation as well as an overview scheme for their expectable "day to day progress" in hospital. The latter should explicitly level the patients' expectation on achievable functional and daily life progress during the first weeks after surgery. Attendance of this personal briefing session was voluntary; each individual was personally by the local study coordinator.

Alongside their social life circumstances the patients were hospitalized on the day of surgery. The elderly as well as patients living in larger distance from the department were hospitalized at the day prior to surgery. Further medical treatment followed standardized milestones. Discharge planning was done on day one after surgery; according the German standards the majority of patients underwent in house rehabilitation after hospital stay.

As a feature of the clinical pathway, "pathway patients" were gathered into groups to have surgery on the same day and to become hosted in the same room(s). The team in the operation theatre was not changed throughout the day and operated 3 - 4 pathway patients. During the indoor post surgical rehabilitation course physiotherapy exercises were done in the patient room(s) within the actual group of "pathway patients" [19,20].

### Patient Characteristics

The patient cohorts before and after pathway implementation showed a median age of 68 versus 69 years at the day of surgery (64% versus 57% females, respectively). Respective fractions of 32% versus 29% of the patients reported to live alone, 8% versus 9% reported a regular working employment (at least 4 hours per day) during a six months period before surgery; 22% versus 17% reported a higher education (academic degree, college etc).

A sub cohort of only 58 patients attended the personal briefing offer as part of the clinical pathway under consideration (Table 1). However, the sub cohorts with and without attendance of the briefing did not differ among each other concerning sociodemographic characteristics or surgical process determinants (Tables 1 and 2, respectively); furthermore they did not differ from the first cohort before pathway implementation.

**Table 1:** Sociodemographic cofactors in TKA patients before and after implementation of a clinical pathway were found comparable.

	before pathway implementation	after pathway implementation, without personal briefing	after pathway implementation and personal briefing
	(n = 132)	(n = 70)	(n = 58)
***age: median and range [years]***	68 (43 - 88) years	69 (46 - 85) years	70 (53 - 80) years

*< 60 years*	19%	20%	16%

*60 - 70 years*	40%	34%	40%

*> 70 years*	41%	46%	45%

***females***	64%	54%	59%

***living alone***	32%	30%	26%

***under employment***	8%	10%	9%

***graduate***	20%	13%	22%

distribution characteristics for sociodemographic cofactors assessed in 132 patients, who underwent total knee arthroplasty (TKA) before implementation of a critical pathway on TKA, versus 128 patients, who underwent TKA after path implementation (the latter being stratified for attendance of a personal briefing as a voluntary part of the pathway)

**Table 2:** Surgical process characteristics in TKA patients before and after implementation of a clinical pathway were found comparable.

	before pathway implementation	after pathway implementation, without personal briefing	after pathway implementation and personal briefing
	(n = 132)	(n = 70)	(n = 58)
***surgery performed by trial investigator***	25%	30%	22%

***median duration of surgery***	88 min	83 min	87 min

***median duration of indoor treatment***	9 days	9 days	9 days

***patients with PCCL***^***1 ***^„***0"***	42%	60%	55%

***patients with PCCL***^***1 ***^„***3" or ***„***4"***	34%	27%	24%

distribution characteristics for surgical process characteristics assessed in 132 patients, who underwent total knee arthroplasty (TKA) before implementation of a critical pathway on TKA, versus 128 patients, who underwent TKA after path implementation (the latter being stratified for attendance of a personal briefing as a voluntary part of the pathway)

^1^PCCL = patient comorbidity complexicity level

### Primary Endpoints and Evaluation Target Parameter

The primary clinical endpoint of this investigation was the individual gain in algo-function as assessed by means of the WOMAC osteoarthritis questionnaire: one week before as well as three months after surgery patients answered the 24 WOMAC items, which were documented in terms of a five-staged ordinal scale. The 24 items were then averaged and transformed into a scale of 0 - 100%, where the scale maximum 100% indicates optimum well-being in each of the 24 items. The intraindividual difference post - pre [%] of this transformed WOMAC index was then considered as a surrogate for the patients' clinical benefit achieved by TKA.

The primary economic endpoint of this investigation were the total direct costs of the health care process [€] from the Orthopedic Surgery Department's perspective as coordinating and cost charging health care provider unit. Total costs were estimated by means of individual sub process cost documentation including the following hospital associated cost segments: anaesthesia, radiology, laboratory, surgery, implant and associated medicinal devices, medication costs, stationary care charges and physiotherapy care charges. Note that this primary estimation did not involve direct human ressource-related costs for the implementation and maintenance of the pathway by the Department members, but only direct costs related to the patient-related intervention constituents such as the written material and the reimbursement of personal briefing attendances.

The primary health economic target parameter of the investigation was then the individual marginal cost effectiveness ratio (MCER) relating a patient's process cost sum to his/her individual gain in algo-function as assessed by means of the WOMAC index [€/%]. In terms of this target parameter the clinical pathway under consideration was considered cost efficient, as soon as the (sub) process costs in the first cohort before pathway implementation were found higher than in the second cohort after implementation; furthermore, the pathway was considered cost effective, if in addition the WOMAC index increase in the second cohort was not lower than in the first cohort and, in addition, the MCERs after pathway introduction showed smaller costs per gained benefit unit.

The confirmatory cohort comparison was based on the MCER, where individual cost effectiveness of a patients' care process was defined by means of a (sub) process cost/patient benefit relation ≤ 100 €/% WOMAC increase. This benchmark was defined a priori of the trial and can be motivated as follows: The German DRG rate amounts to about 7500 € for TKA, which can be expected to result in median WOMAC increases of about 40%. The ratio of these median estimates would then become 7500 €/40% = 188 € per gained % increase in the WOMAC index due to TKA. Bearing this heuristic benchmark in mind, individual costs of 100 € per gaines WOMAC unit would correspond to a quite cost efficient health care process. However, since the DRG rate covers any financial investment from the hospital's perspective, it corresponds to a *maximum *cost sum from the health care providers' perspective. Bearing the above sub process components in mind, the authors pre-calculated an expectable sub process cost sum of 4500 €, which was hoped to reduce to 4000 € after clinical pathway implementation. The corresponding MCERs would become 113 € versus 100 € per gained % WOMAC, respectively. In accordance to these heuristic median estimates, an individual MCER estimate ≤ 100 €/% was defined as an indication for "individual cost effectiveness" for the respective patient's health care (sub) process.

### Statistical Analysis and Sample Size Consideration

The confirmatory analysis of this cohort investigation was based on an exact two-sided Fisher test at the 5% significance level to compare the frequencies of "individually cost effective" care between the cohorts before and after clinical pathway implementation, it exceeds the relative frequencies of patients showing a cost/benefit relation ≤ 100 € per % WOMAC increase. In the planning phase of the investigation, a frequency of at least 75% individually cost effective processes was expected before versus at least 90% after pathway implementation. To achieve a statistical power of at least 80% in the detection of this assumed difference by means of an exact Fisher test at the two-sided 5% level, a minimum net cohort size of 113 patients had to be recruited.

The distributions of continuous endpoints such as the primary clinical endpoint and the MCER were described by medians and quartiles (graphically on nonparametric box plots, accordingly) to take account for possible statistical outliers. The description of categorical endpoints was based on absolute and appropriate relative frequencies. These methods were applied to the overall sample as well as to sub samples. Intraindividual comparisons were based on the description of difference distributions for continuous endpoints and on total frequencies in contingency tables for categorical endpoints.

The significance evaluation of intraindividual changes in continuous endpoints was based on sign tests, sub sample comparisons were based on pairwise Wilcoxon two sample tests and on pairwise Fisher tests for categorical endpoints. Results of these tests were summarized in terms of p-values. Due to the rather exploratory character of the sub sample comparisons, these p-values were not formally adjusted for multiplicity. A p-value < 0.05 therefore indicates locally significant sub sample differences.

A multivariate re-analysis of the cost effectiveness data was based on the MCER cutpoint 100 €/% and the corresponding definition of individually cost effective treatment. This exploratory endpoint allowed for multiple logistic regression modelling of simultaneous cost effectiveness determinants like age, gender, working and family state. Logistic regression modelling was performed by means of Likelihood Ratio tests (foreward selection at a local 5% significance level). All numerical and graphical evaluations were based on the software SPSS^® ^(release 12.0 for Windows^®^).

## Results

### Cohort Comparison

The first cohort before pathway implementation showed a median WOMAC increase of 39% (interquartile range 29 - 48%) from 41% before surgery to 83% three months later in median versus an increase of 35% (24 - 45%) from in median 44% to 82% in the second cohort after pathway implementation (Wilcoxon p = 0.120). The respective sub process sums were estimated 4303 € (4130 - 4660 €) versus 4194 € (4039 - 4429 €; Wilcoxon p = 0.002). Despite this locally significant decrease in costs from the providers' perspective, the individual cost/benefit relation became worse after introduction of the pathway: the median MCER in the first cohort was estimated 108 € per gained% WOMAC index increase (86 - 150 €/%) versus 118 €/% WOMAC gain (93 - 173 €/%) in the second cohort after pathway implementation (Wilcoxon p = 0.367). An individual cost/benefit relation ≤ 100 €/% was found in 38% versus 30% of the respective cohort patients (Fisher p = 0.278); accordingly the introduction of an interdisciplinary clinical pathway on TKA did not significantly change the fraction of individually cost effective treatment courses from the health care providers' perspective in terms of this primary cost effectiveness parameter.

### Sub Cohort Analysis

Stratification of the second patient cohort after pathway implementation further illustrated the above tendency (Table 3): patients who additionally attended the voluntary preoperative patient information/education (n = 58), showed a median WOMAC change of 30% (21 - 45%) from 46% in median before to 83% after surgery, versus a median increase of 38% (30 - 47%) from 44% to 82% among patients without patient information/education (n = 70). The sub cohort of patients having attended a personal briefing, as part of the implemented clinical pathway, significantly differed from the first patient cohort before pathway implementation (median WOMAC increase 39%, Wilcoxon p = 0.029).

**Table 3:** WOMAC index and sub process cost sum distributions {€] for TKA changed after the introduction of a clinical pathway.

	before pathway implementation	after pathway implementation, without personal briefing	after pathway implementation, including personal briefing
	(n = 132)	(n = 70)	(n = 58)
***WOMAC index before surgery [%]***	**41%** (32; 48%)	**44%** (34; 50%)	**46%** (39; 54%)

***WOMAC index three months after surgery [%]***	**83%** (68; 91%)	**82%** (74; 91%)	**83%** (66; 90%)

***intraindividual three months change in WOMAC [%]***	**39%** (27; 48%) range -20; 69%	**38%** (30; 47%) range -6; 68%	**30%** (21; 45%) rrange +1; 71%

***sub process cost sum [***€***]***	**4303 **€ (4130; 4660 €)	**4149 **€ (3980; 4443 €)	**4244 **€ (4097; 4412 €)

***individual cost effectiveness ratio [***€***/% WOMAC change]***	**108 **€ **/%** (86; 150 €/%)	**110 **€ **/%** (88; 145 €/%)	**135 **€ **/%** (102; 211 €/%)

***individual effect costs [% WOMAC change/1000 ***€***process cost investment]***	**9%/1000 **€ (6; 11%/1000€)	**9%/1000 **€ (7; 11%/1000€)	**7%/1000 **€ (5; 10%/1000€)

medians and quartiles for the total WOMAC osteoarthritis index [%, 100% = optimum rating] before and three months after total knee arthroplasty (TKA) as well as intraindividual post - pre change [%] of the index, sub process cost sum {€] for TKA from the hospital's perspective as well as individual ratios between the latter (cost effectivemess, [€/%] and effect costs [%/1000 € investment], respectively), assessed in 132 patients, who underwent TKA before implementation of a critical pathway on TKA, versus 128 patients, who underwent TKA after path implementation (the latter being stratified for attendance of a personal briefing as a voluntary part of the pathway)

The sub cohorts after pathway implementation showed median process cost sums of 4149 € (3980 - 4430 €, without briefing) versus 4244 € (4097 - 4412 €, with briefing; Wilcoxon p = 0.230) and median MCERs of 110 € versus 135 € per gained% WOMAC increase (Wilcoxon p = 0.013). According to the locally significant difference in clinical outcome, the sub cohort of patients who attended the patient information/education significantly differed from the first cohort before pathway implementation concerning the cost/benefit relation (Wilcoxon p = 0.005), but not concerning the process cost sum (Wilcoxon p = 0.074). The sub cohort of patients without patient information/education, however, showed a locally significant cost reduction (median cost sums 4149 € versus 4303 €, Wilcoxon p = 0.001) in comparison to the first cohort before pathway implementation, but no difference concerning clinical outcome (median WOMAC increase 38% versus 39%, Wilcoxon p = 0.650). The latter cohorts did not significantly differ in individual cost effectiveness (median MCER 110 € versus 108 € per gained% WOMAC increase, Wilcoxon p = 0.936).

Accordingly, in terms of the primary endpoint of this evaluation, the sub cohorts with and without attendance to the pathway-associated briefing showed 36% versus 23% individually cost effective patient courses (individual MCER ≤ 100 €/%); however; the two sub cohorts after pathway implementation did not signficantly differ in this primary cost effectiveness endpoint (Fisher p = 0.166).

### Multivariate Analysis

Logistic regression modelling of the exploratory endpoint "individual MCER < 100 €/% WOMAC increase" confirmed the dominating sub cohort impact on the cost effectiveness outcome: neither age, gender nor working or family state of the patients were found to be significantly associated with the occurrence of individually cost effectiveness in terms of an MCERs ≤ 100 €/% (Likelihood Ratio p = 0.200, p = 0.675, p = 0.730 and p = 0.205, respectively). However, in contrast to the univariate findings, no statistical signficance of the sub cohort affiliation could be reproduced by logistic regression modelling of this binary cost effectiveness endpoint (Likelihood Ratio p = 0.493 for the contrast between the first cohort before and the briefed sub cohort after pathway implementation, p = 0.705 for the corresponding contrast between the respective sub cohorts after pathway implementation with and without briefing).

The only putative explaining variable, which was found significantly associated with the binarized MCER endpoint, was the patients' preoperative assessment of their algo-functional status (Likelihood p < 0.001 for the preoperative WOMAC index). This impact of the preoperative WOMAC level corresponds to the univariate cohort gradient in median preoperative WOMAC levels (41% before versus 44% and 46% after pathway implementation). It is illustrated by Table 4: patients with a preoperative WOMAC index > 40% showed individually cost effective process courses in terms of an MCER ≤ 100 €/% in 52% (first cohort) versus 56% and 47% (sub cohorts without and with briefing after pathway implementation, respectively). In summary, the observed sub cohort difference in cost effectiveness can be explained as an epi-phenomenon of a preoperative gradient in algo-functional status among the respective cohorts. Note, however, that the goodness of model fit was only moderate for these evaluations with an overall Nagelkerke coefficient of only 68%.

**Table 4:** Individual cost effectiveness ratios alongside a critical pathway on TKA are associated with algo-functional status before surgery

		individual cost effectiveness	individual cost effectiveness
	*WOMAC index [%] before TKA*	≤***100 ***€***/%***	***> 100 ***€***/%***
***before pathway implementation***			

	≤ *40%*	25%	75%

	*> 40%*	52%	48%

***after pathway implementation, without personal briefing***			

	≤ *40%*	24%	76%

	*> 40%*	56%	44%

***after pathway implementation and personal briefing***			

	≤ *40%*	13%	87%

	*> 40%*	47%	53%

relative frequencies of individual cost effectiveness ratios ≤ 100 €/% between partial cost sum {€] from the hospital's perspective and three months WOMAC change after TKA [%], assessed in 132 patients, who underwent TKA before implementation of a critical pathway on TKA, versus 128 patients, who underwent TKA after path implementation (the latter being stratified for attendance of a personal briefing as a voluntary part of the pathway), stratified for the patients' total WOMAC osteoarthritis index before surgery [„≤ 40%" versus „> 40%"]

## Discussion

This study intended to evaluate the effects of a clinical pathway for total knee arthroplasty in terms of patient-related (self-reported) daily life function and process cost profiles from the health care providers' perspective by means of a prospective pre-post cohort evaluation. In summary, the introduction of an interdisciplinary clinical pathway did not increase patient related outcomes in terms of the confirmatory outcome assessment (WOMAC total index); on the contrary, during univariate confirmatory analysis the cost effectiveness from the health care providing Orthopedic Surgery Department's perspective was even found reduced. By means of multiple logistic regression analysis, the underlying cohort difference in cost effectiveness could be partially explained as an epi-phenomenon of a preoperative gradient in algo-functional status among the respective cohorts before and after pathway implementation; it may therefore be considered as having arisen from a somewhat asymmetric regression to the mean effect within the sub cohorts due to a slight (non significant) sub cohort difference in the preoperative WOMAC scores. Nevertheless, the recently (13) well-emphasized positive effect of clinical pathways on both economic and clinical outcome parameters could not be reproduced within the above sequential cohort design investigation.

### Design Considerations

The sequential cohort design was considered as an alternative to a (cluster) randomized trial on the effect of the pathway under consideration: note that the parallel conduction of a "pre-pathway" cohort and a "pathway cohort" within the same department will hardly be possible due to uncontrollable mechanisms of cohort interactions and thereby improvement of process quality in the "pre-pathway" cohort in the direction of the pathway under investigation. A severely biased cohort difference (if any) would be the result of such a randomized investigation, i.e. the benefit of its higher evidence level shall become crucially reduced by these sources of bias. The same holds for parallel (cluster randomized) cohorts, which would afford involvement of several Orthopedic Surgery departments to avoid the bias mentioned above. Such a trial design, however, might introduce severe bias due to the locally differing surgical investigators within the trial sites' pathway realisations. The observed "pre-" versus "post-pathway" difference would then become confounded by cofactors rather related with the local orthopedic surgeons and their implant preferences than with the underlying pathway proposals. In summary, despite its inferior level of evidence, the sequential cohort design was considered less biased for the recent research intention.

Note, however, that the design at hand may have introduced a different kind of bias due to the choice of time for the main intervention: the clinical pathway proposal under consideration mainly concentrates on patient-related information by means of written material and personal briefing; it thereby addresses the area of patient compliance and awareness. As a matter of fact, this central part of the intervention was implemented before (!) the preoperative WOMAC assessment of the post pathway cohort, i.e. the preoperative functional assessment of this post-intervention cohort may have been biased in contrast to the corresponding ratings in the pre-intervention cohort. This bias may then also have been introduced into the marginal cost effectiveness estimates and thereby into the primary endpoint evaluation. On the other hand, Table 3 indicates, that all three sub cohorts showed comparable preoperative median WOMAC scores (not differing locally significantly), with preoperative medians increasing proportionally to the amount of individually achieved information. Whether this gradient in preopative index distributions is rather an indication of intervention-related bias in the above meaning or rather represents a consequence of different recruitement patterns among the pre and post pathway implementation cohorts (related to the willingness to attend briefings) cannot be quantified by means of the data at hand.

Furthermore, one must critically discuss the fact that the attendance of the personal briefings as a central constituent of the pathway intervention was held voluntary: note that only 58 out of 128 patients accepted this offer and thereby put the pathway under consideration into question! This could have been and, in fact, was expected during the planning phase of the investigation with regard to the patients age, clinical and socioeconomic status: note that TKA patients usually will require the help of relatives (to provide a lift etc) to attend personal briefing schedules. Nevertheless, we found it unethical to force our trial participants to attend the briefings "by all means" (which, in addition, might again have introduced severe bias into recruitement patterns and preoperative ratings!).

### Clinical Considerations

The interdisciplinary conception of the clinical pathway for TKA was based on medical records and data from an initial prospective cohort [1,2]. Prior to this study the treatment for total hip arthroplasty was organized by means of a clinical pathway by the same Orthopaedic Surgery department. As a potential side effect of the resulting pathway experience within the overall department, the first TKA cohort ("pre-pathway") already underwent rather elaborate standards, which may have implicitly arisen from the previous THA pathway experience: for example, all patients undergoing total joint arthroplasty were seen 5-7 days prior to surgery to check the medical condition and the completeness of medical records; furthermore their LOS was standardized and optimized. As a putatively implicit consequence, median and quartile length of stay for total knee arthroplasty patients were both already reduced to 9 days in the first cohort before explicit definition and implementation of the TKA critical pathway. Bearing these initial conditions in mind, the rather moderate effect of the above pathway introduction on process characteristics such as LOS becomes motivated by the following facts from literature:

a) failing of improvement in cost effectiveness for patients undergoing TKA was also found in other studies [21,22]

b) like in our study no further improvement in patient-related outcome was found after accelerated intervention [22]

c) the potential of clinical pathways to show sequential improvement in patient care and cost reduction seems obviously limited, as quantified by Vanhaecht by means of repeated observations of sequentially modified TKA pathways [11,14]

In this sense the previous implementation of a clinical pathway for total hip arthroplasty in the investigators' hospital might already have implied an implicit pathway optimization onto TKA processes as well, before any "pre-pathway" data documentation was started in the first cohort. In summary the main interventional constituents of the clinical pathway under consideration here were "patient information/education", "grouping of patients during physiotherapy and hospital stay", "securing the treatment safety with milestones and discharge planning" and, in particular, "leveling patient expectation by explaining expectable postoperative day-to-day progress" as well as "preoperative training in the postoperative use of walking aids". No interventions concerning the implant were made. All patients received the same implant at the same costs [9,23].

The observed total order improvement in the WOMAC score for patients undergoing total knee arthroplasty is comparable to other studies [9,23-26]. Nevertheless, the somewhat smaller median increase in the second sub cohort (median increase 30% versus 39% and 38% in the other sub cohorts, respectively), affords critical consideration: the patient information/education were offered to all patients of the second cohort. The sub cohort of patients, who accepted these offers was found higher educated and showed an overall better medical condition as compared to the cohort refusing this offer (Tables 1 and 2). The baseline WOMAC index of the latter sub cohort was found higher, but its three months WOMAC index ended at the same level than did the other cohort's median index. As a consequence the median intraindividual WOMAC changes turned out smaller for the "highly informed" sub cohort. It is yet unclear whether this effect is a result of the information offer and an increased expectation within the informed sub cohort [27] or rather a patient-associated selection bias in the direction of a better preoperative health condition (Table 2) as a surrogate of the ability to attend the personal briefings.

### Economic Considerations

The screened costs from the health care providers' perspective were found significantly lower in the second cohort. In contrast to other studies this cost reduction was not achieved by shortening the length of stay or change in implant selection [8,9,11,28,29]: consideration of the cost components indicated that grouping of patients for physiotherapy had contributed the main part to the observed overall cost reduction. In contrast to this observation, most authors found maximum cost reduction from the health care providers' perspective by reducing or at least standardizing the LOS: the potential to show an improvement in cost-effectiveness by implementation of critical pathways depends on the initial medical treatment. However, our initial LOS for patients undergoing total knee arthroplasty appears to be short compared to the German population of patients undergoing TKA and other European groups [11,30]. As a consequence from both an ethical and clinical point of view, the clinical project investigators were - with respect to patient safety - unwilling and unable to implement a further shortening in LOS, and thereby the individual cost effectiveness ratios turned out merely unchanged for the cohort, who refused the offered patient information/education.

On the other hand the variation in observed process cost sums declined for this cohort, which indicates an intrinsic benefit of the pathway implementation in terms of process costs as one main target in clinical pathways [31].

Nevertheless, bearing this locally significant cost reduction after pathway implementation in mind, a different source of human resources related costs must be critically discussed: the above presentation primarily considered direct process costs for health care from the hospital's perspective, which meant concentration on investment for treatment and care as well as on human resources necessary to provide the latter. A future stage of this evaluation will concentrate on a cost-cost analysis and estimate the human resource investment for conception, implementation and maintenance of the critical pathway itself. As a broad initial estimation the latter will amount to not less 25.000 € per year and pathway (and certainely more during the conception and implementation phase), and will thereby crucially reduce the nominal cost reductions described above.

## Conclusions

In terms of a cost-cost balance, surgical department coordinators may feel warned, that the implementation of critical pathways presents an investment into quality, but - at least after already having achieved a certain stage of process quality - may not necessarily result in cost reduction, but can even end up in increased human resource investment. Furthermore one should note, that it cannot be avoided that a critical pathway will involve more of less specific local conditions, which will make it more explicit and easier to apply for local residents, but require immediate and dynamic changes of the pathway as soon as these local conditions change or are being changed: if, for example, local physiotherapy staff is no longer directly available due to structural changes in the overall hosting University Hospital, the less direct accessibility of physiotherapy within the critical pathway's course may require its complete redesigning.

To our understanding a clinical pathway will not serve as a "magic wand" to induce unlimited improvement in quality of care and cost saving. Clinical pathways represent a management tool to organize and optimize patient care by assuring maximum possible and consistent medical quality and cost containment. Within already optimized or at least well-organized medical circumstances a clinical pathway is unable to further improve the cost-effectiveness of treatment courses. Nevertheless, pathway screening and maintenance as a tool of continuous quality management can still be used to ensure the achieved level of medical and economic process and outcome quality.

Further research on this topic is certainly needed [14,15,31], in particular with regard to the increasing number of patients awaiting TKA and THA within the forthcoming decades.

## Competing interests

**Financial competing interests/Funding**: The investigation was granted by Zimmer^® ^GmbH Deutschland: the study coordinator position of Ms Claudia Wolf was sponsored by this company grant to enable personal patient interviews, documentation of the results presented in this manuscript and for assistance in derivation and implementation of the clinical pathway under consideration; furthermore the Zimmer company grant comprised the expenditures for patient information booklets as well as for statistical analysis of the patient and cost data presented here.

The authors assure the absence of any individual or employer-related financial, political or non-financial competing financial interest.

## Authors' contributions

The corresponding author (FK) has designed the investigation at hand (calculation of sample size, choice of endpoints and target parameters, choice of sequential cohort design etc), designed and implemented the statistical analysis of the cohort data (including development of the investigation's Statistical Analysis Plan and writing of the full biometrical report as part of the statistical analysis), thoroughly supervised the medical documentation project staff, wrote the initial manuscript draft in full version and implemented all revisions.

The second author (KPG) took part in the trial designing discussion, established the communication with the company Zimmer Germany to enable financial funding of the project by this partner, performed surgery on trial patients and revised the initial manuscript draft from a clinical perspective.

The third author (SK) took part in the trial designing process from a clinical perspective (choice of clinical endpoints and economic cost parameters), recruited and performed surgery on trial patients, added comprehensive parts of the manuscript from a clinical perspective, co-supervised the medical documentation project staff and supervised the medical project staff.

All authors (FK, KPG and SK) have read and approved the full manuscript in both its initial and final version.

## Pre-publication history

The pre-publication history for this paper can be accessed here:

http://www.biomedcentral.com/1472-6963/11/338/prepub
